# A rare 3q13.31 microdeletion including *GAP43* and *LSAMP* genes

**DOI:** 10.1186/1755-8166-6-52

**Published:** 2013-11-26

**Authors:** Stefania Gimelli, Massimiliano Leoni, Maja Di Rocco, Gianluca Caridi, Simona Porta, Cristina Cuoco, Giorgio Gimelli, Elisa Tassano

**Affiliations:** 1 Service of Genetic Medicine, University Hospitals of Geneva, Geneva, Switzerland; 2 USD Malattie Rare, Istituto G Gaslini, Genoa, Italy; 3 Laboratorio di Fisiopatologia dell’Uremia, Istituto G. Gaslini, Genoa, Italy; 4 Laboratorio di Citogenetica, Istituto G. Gaslini, Genoa, Italy

**Keywords:** 3q31.31microdeletion, *GAP43* gene, *LSAMP* gene, Genotype-phenotype correlation

## Abstract

**Background:**

Interstitial deletions affecting the proximal long arm of chromosome 3 have been rarely reported in the literature. The deleted segments vary in localization and size with different breakpoints making genotype-phenotype correlation very difficult. Until now, a girl with a 1.9-Mb interstitial deletion of 3q13.2q13.31 and 14 novel patients with deletions in 3q11q23 have been reported.

**Results:**

Here we report on a 7-year-old girl with neuropsychiatric disorders and renal, vascular and skeletal anomalies. Array-CGH analysis revealed a small rare inherited 3q13.31 deletion containing only two genes, *GAP43* and *LSAMP*. The mutation analysis of the two genes was negative on the other non-deleted chromosome. *GAP43* is considered a crucial component for an effective regenerative response in the nervous system and its mRNA is localized exclusively to nerve tissue where the protein is linked to the synaptosomal membrane. *LSAMP* is a 64- to 68-kD neuronal surface glycoprotein found in cortical and subcortical regions of the limbic system that acts as an adhesion molecule and guides the development of specific patterns of neuronal connection. The deleted region is adjacent to a “desert gene” region extending 2.099 Mb.

**Conclusions:**

We discuss the effects of *GAP43* and *LSAMP* haploinsufficiency, proposing that their deletion may be responsible for the main phenotype. Further cases with similar microdeletion are expected to be diagnosed and will help to better characterize the clinical spectrum of phenotypes associated with 3q13.31 microdeletion.

## Background

Interstitial deletions of the proximal long arm of chromosome 3 are quite rare. In addition, the deleted segments vary in localization and size and have different breakpoints, making genotype-phenotype correlation very difficult.

A girl with a 1.9-Mb interstitial deletion of 3q13.2q13.31 presenting with dysmorphic features, muscle hypotonia, and developmental delay was reported [1]. More recently, 14 novel patients with deletions in 3q11q23 were investigated and compared with 13 previously reported patients [2]. The reported deleted segments were very different in length, spanning from 580 Kb to 22.4 Mb and covering the region 3q12.3q21.3. The same authors indicate, among others, *GAP43* and *LSAMP* as strong candidate genes for developmental delay. GAP43 is involved in neurite outgrowth, neurotransmission, and synaptic plasticity. It has been recently identified as a candidate gene for autism and autistic-like manifestations in humans and mice [3-5].

*LSAMP* gene gives rise to LAMP, which is a 64- to 68-kDa heavily glycosylated protein, structurally characterized by three immunoglobulin (Ig) domains [6]. LAMP protein proved to be specific to the cortical and sub-cortical limbic-associated regions, but it has been found less intensely in the midbrain and hindbrain of the developing and adult brain [7]. Studies in both humans and mice have demonstrated the involvement of *LSAMP* in neuropsychiatric features and behaviour [8-11].

Here, using array-CGHanalysis, we have identified a small rare inherited 3q13.31 deletion containing only two genes (*GAP43* and *LSAMP*) in a girl with neuropsychiatric disorders associated to renal, vascular, and skeletal anomalies.

## Case presentation

**Figure 1:**
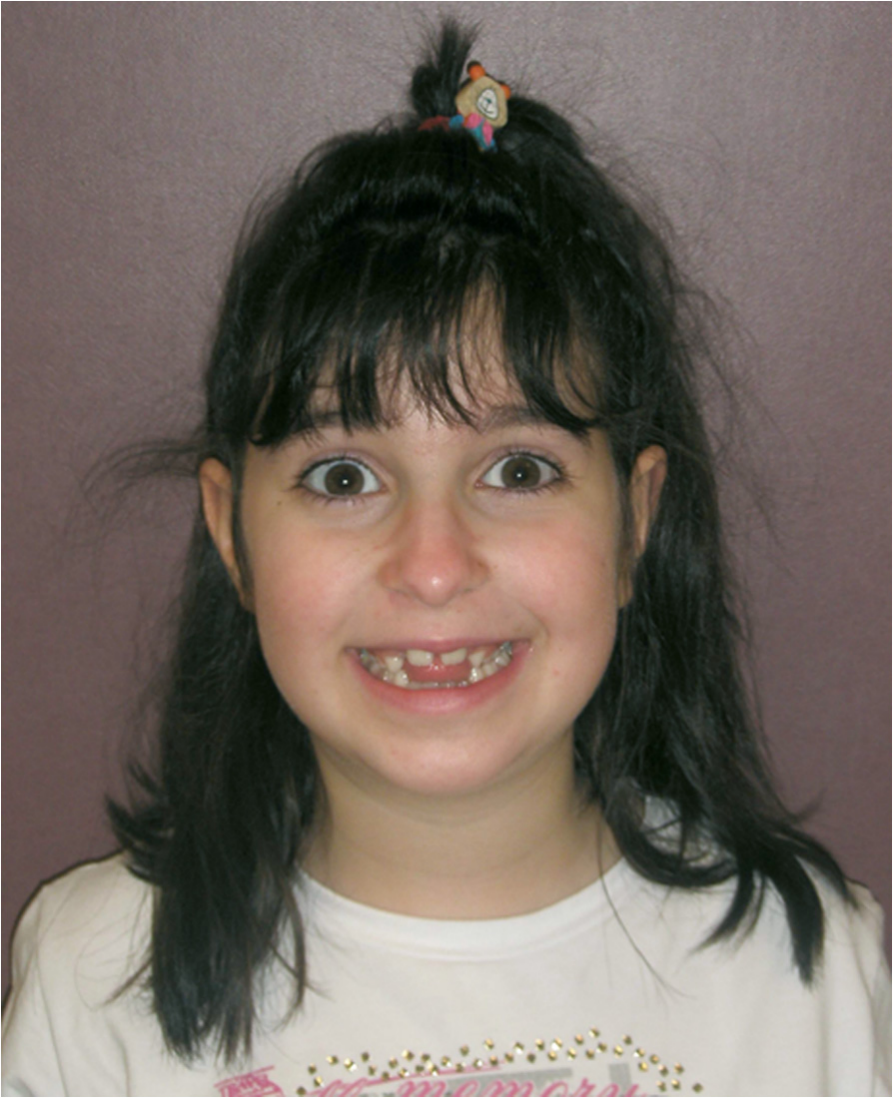
The patient.

A 7-year-old girl (Figure 1) was referred to our hospital because of developmental delay and clumsiness. Her father had slightly delayed psychomotor development but his cognitive level was never tested. The patient was born at term to unrelated healthy parents after an uneventful pregnancy. At birth, weight and length were normal, 3580 g and 47 cm, respectively. Postnatal overgrowth was evident: weight was 38 kg (90th-97th centile), and height 130 cm (75th-90th centile). Distinctive facial features like tented upper lip and arched palate were present, too. Although no delay in reaching psychomotor development milestones was reported, she presented behavioural immaturity, clumsiness, motor incoordination, and attention deficit. Moreover, muscular examination showed hypotonia. Jumping and deambulation were clumsy, in particular walking on heels and on tiptoes. Electroencephalography revealed paroxysm in the temporal region, with no seizure-related pattern nor history of seizures. Abdominal ultrasonography revealed a small left kidney, with puckered appearance, while the right kidney was increased in size to compensate for the malformation. Renal scintigraphy showed 4% residual function. The abdominal ultrasonography also showed dilated left epigastric vein, evident on abdominal wall. Subsequent magnetic resonance angiography revealed inferior vena cava agenesis. Common iliac veins were absent, too. Collateral venous circulation was identified via ectasic paravertebral veins and azygos-hemiazygos vein system. Furthermore, hypoplasia of left external iliac, popliteal and femoral veins was showed. The left renal artery coul not be visualized. Finally, the patient presented skeletal malformations, in particular: left leg longer than right leg, flat feet, hypoplastic right lower limb, and femoral varus deformity.

**Figure 2:**
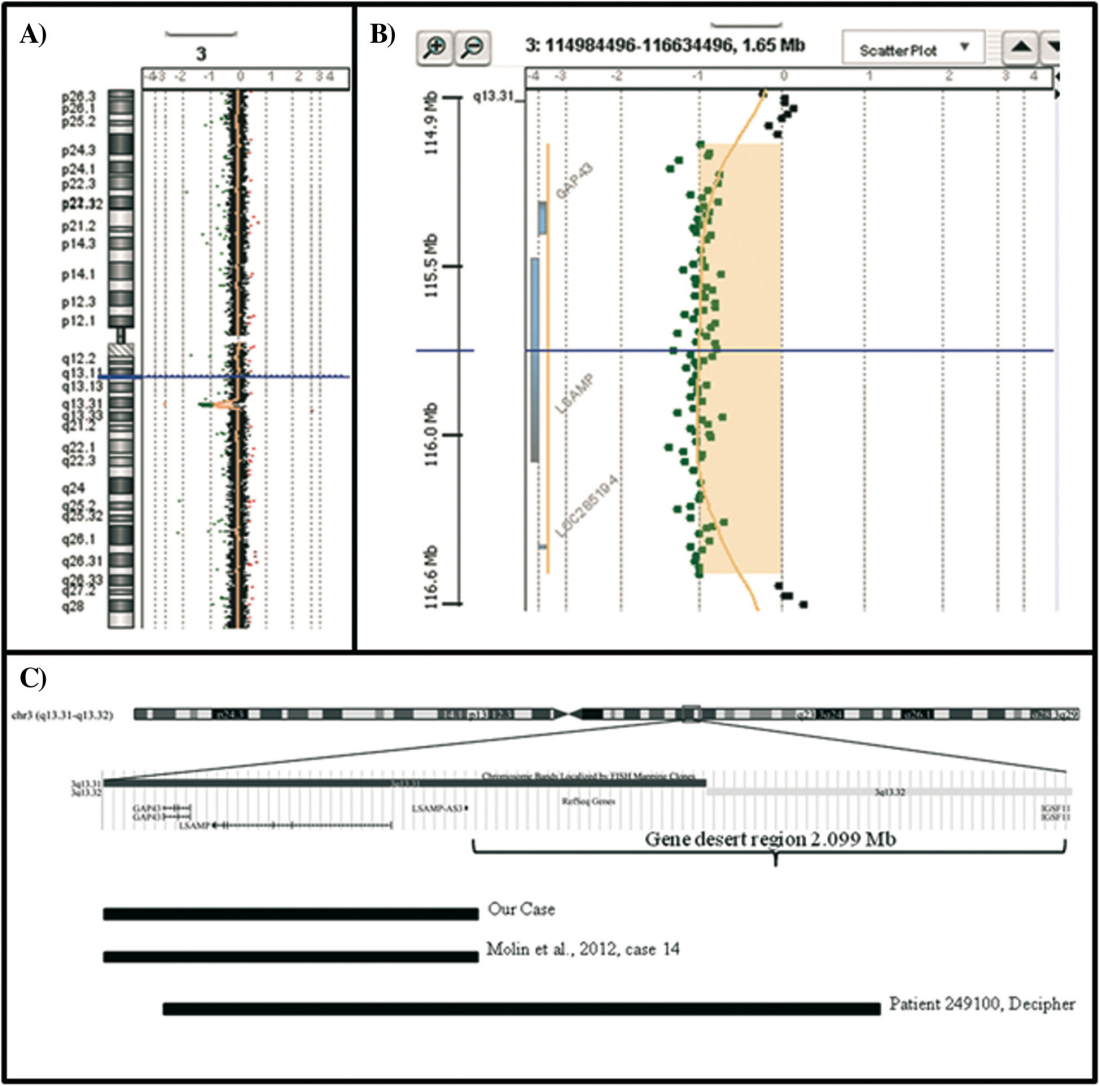
**Results of Array-CGH analysis. A)** Ideogram and **B)** Ratio plot of array-CGH analysis show the presence of 1.362 Mb deletion at 3q13.31 band with breakpoints at genomic positions 115,157,887 bp and 116,520,120 bp (GRCh37/hg19, Feb 2009). **C)** Extract from the UCSC genome browser (http://genome.ucsc.edu/) GRCh37/hg19 shows the deleted region and its gene content compared to the deletions identified by Molin et al. [2] and by DECIPHER database.

Conventional cytogenetic analysis of the proposita and her parents showed a normal karyotype. In the absence of an etiological diagnosis an array-CGH analysis using Human Genome CGH Microarray Kit G3 180 (Agilent Technologies, Palo Alto, USA) with ~13 Kb overall median probe spacing, was performed. Array-CGH analysis identified a 1.362 Mb deletion at 3q13.31 band with breakpoints at genomic positions 115,157,887 bp and 116,520,120 bp (GRCh37/hg19, Feb 2009) (Figure 2A, B). The deleted region is adjacent to a “desert gene” region extending 2.099 Mb (chr3:116,520,120-118,619,479) (Figure 2C). The deletion was inherited from the father and contains only two genes, *GAP43* (MIM 162060) and *LSAMP* (MIM 603241), and a long non-coding RNA (LSAMP-AS3) (Figure 2C). The region is not covered by benign CNVs (http://dgv.tcag.ca/dgv/app/home). Mutational screening of *GAP43* and *LSAMP* performed by bi-directional Sanger sequencing of exons and flanking introns, was negative on the homologous allele.

Recently, Molin et al. [2] reported on 14 patients with a novel microdeletion syndrome at 3q13.31 characterized by developmental delay, postnatal overgrowth, hypoplastic male genitals, and facial dysmorphisms. The deleted region spanned from 580 Kb to 22.4 Mb with different boundaries. Among them only one patient (patient 14) carried a deletion of 1.176 Mb with an uncertain inheritance (absent in the mother and father not tested) similar to our case. This deletion involved *GAP43* and *LSAMP* genes but unfortunately a not better specified “developmental delay” was the only reported phenotypic feature concerning the patient. A second overlapping case is described in DECIPHER database (Patient 249100), as affected by behavioural problems, constipation, mental retardation/developmental delay, and microcephaly.

GAP43 is found in growth cones of extending axons in the central nervous system [12,13]. It has many functions including growth cone navigation, neurite outgrowth, stabilization of axonal branches, neurotransmission, and synaptic plasticity [4]. Mice lacking one allele for *GAP43* show multiple failures to establish or maintain long-distance cortical connections. *GAP43* was also recently identified as a candidate gene for autism and autistic-like manifestations in humans and mice [3-5] and Gap43^+/-^ heterozygous mice displayed decreased corpus callosum and hippocampal commissure volume [5], as also highlighted by Molin et al. [2].

*LSAMP* encodes the limbic system-associated membrane protein, and studies in both humans and mice have demonstrated the involvement of *LSAMP* in neuropsychiatric features and behaviour [8,9].

In addition to a neuropsychiatric phenotype, our patient presented renal, vascular, and skeletal anomalies. Interestingly, *LSAMP* is also expressed in kidney [14], in cardiovascular tissues in humans [15] and in mice (MGI database), and in osteoblasts [16,17]. Moreover, *GAP43* is also expressed in mouse renal-urinary system (MGI database) and in skeletal muscles [18].

Several aspects may account for the phenotypic variability described among carriers of microdeletions/microduplications, including variation in genetic background, epigenetic phenomena such as imprinting, expression or regulatory variation among genes in the rearrangement region, and (in the case of deletions) the unmasking of recessive variants residing on the single remaining allele. In our patient, we excluded the presence of mutations in the homologous alleles of *LSAMP* and *GAP43* genes. However, we cannot exclude the possibility of a variable expressivity or an incomplete penetrance, since the father of our patient showed a mild neurological phenotype.

## Conclusions

In conclusion, we have identified a patient with a deletion in 3q13.31 chromosome band containing only two genes (*LSAMP* and *GAP43*) inherited from his father. No mutation was present on the homologous alleles. The father had slightly delayed psychomotor development. We suggest that *LSAMP* and *GAP43* genes are the most likely candidate genes for the phenotypic core of our patient. Nevertheless, the description of more patients with similar microdeletions would be useful to further delineate the main clinical features and to understand the function of the genes contained in the deleted chromosomal interval.

## Consent

The current study was performed using peripheral blood of the members of the family treated at the Istituto Giannina Gaslini, Genova, Italy. The parents of the patient gave written informed consent allowing molecular and genetic studies. We did not request approval by our Institutional Review Board, because our study required only classical and molecular cytogenetic analyses. For cytogenetic analyses only written informed consent of the parents (DM 21 dicembre 2007) is sufficient. The informed consents of the parents were previously authorized by our Institutional Review Board. We did not conduct research outside our country of residence. We did not approach the local authorities before beginning work on this study. The full name of the Ethics Committee of our institution is Comitato di Etica per la Ricerca Scientifica Biomedica, per la Buona Pratica Clinica e per la Sperimentazione dei Farmaci. The parents of the patient allowed us to publish the descriptive details of their children's malformations.

The parents of the individual in this manuscript have given written informed consent to publish these case details. We have the consent from the parents to publish photographs.

### Clinical material

The current study was performed using peripheral blood of the patient and her parents treated at the Istituto Giannina Gaslini, Genova, Italy.

## Competing interests

The authors declare that they have no competing interests related to this manuscript.

## Authors’ contributions

SG, SP, CC, GC, GG, ET have made substantial contributions to conception and design, acquisition of data, analysis and interpretation of data. ML, MD, CC, GG, ET have been involved in drafting the manuscript and revising it critically for important intellectual content. All authors read and approved the final manuscript.
